# Strategies to Close the PrEP Uptake Gap Among Transgender People and Men Who Have Sex with Men in Tshwane, South Africa: Perspectives from the Community

**DOI:** 10.1007/s10461-024-04300-7

**Published:** 2024-03-01

**Authors:** India Perez-Urbano, Athmanundh Dilraj, Annah Pitsi, Naomi Hlongwane, Nada Abdelatif, Janan Dietrich, Khatija Ahmed

**Affiliations:** 1 University of California San Francisco School of Medicine, San Francisco, CA 94143 USA; 2 Setshaba Research Centre, Tshwane, Soshanguve South Africa; 3 Biostatistics Research Unit, South African Medical Research Council, Cape Town, South Africa; 4 Perinatal HIV Research Unit, University of Witwatersrand, Johannesburg, South Africa; 5 African Social Sciences Unit of Research and Evaluation (ASSURE), Wits Health Consortium, Johannesburg, South Africa; 6 Health Systems Research Unit, South African Medical Research Council, Bellville, South Africa; 7 Department of Medical Microbiology, University of Pretoria, Tshwane, South Africa

**Keywords:** Men-who-have-sex-with-men, MSM, Transgender, Pre-exposure prophylaxis, PrEP, HIV Prevention

## Abstract

HIV Pre-exposure Prophylaxis (PrEP) uptake among transgender (TG) people and gay men and other men who have sex with men (MSM) remains low, despite South Africa being the first African country to approve PrEP. This mixed-methods study used a two-phase explanatory sequential design: (1) quantitative analysis of cross-sectional surveys followed by (2) qualitative in-depth interviews. This study explored facilitators and barriers to PrEP uptake to identify strategies to increase utilization in these key populations. We conducted 202 cross-sectional surveys and 20 in-depth interviews between July 2021 and March 2022 in Soshanguve, Tshwane, Gauteng. Quantitative data were analyzed using univariate logistic regression; thematic analysis was performed for qualitative data. Findings show high willingness to use PrEP but low PrEP uptake. We outline strategies to facilitate PrEP use: (1) demystify daily PrEP by deploying community-engaged PrEP education campaigns; (2) capitalize on existing peer networks; and (3) expand accessible and culturally responsive PrEP service delivery models. We provide feasible recommendations to close the PrEP uptake gap in these key populations in South Africa.

**Supplementary Information:**

The online version contains supplementary material available at 10.1007/s10461-024-04300-7.

## Introduction

Sexual and gender minorities continue to be significantly more vulnerable to acquiring HIV. Globally, gay men and other men who have sex with men (MSM) account for 23% of new HIV infections and transgender (TG) people are 13-times more likely to acquire HIV than the general population [1]. South Africa carries the greatest proportion of the global HIV burden with an estimated national prevalence of 13.9% among the entire population [2], 29% (3.1% incidence) among MSM [3], and 45.5 − 63.4% among transgender women [4]. Pre-exposure prophylaxis (PrEP) is a safe and effective HIV prevention tool with impressive population-level impact on HIV incidence when uptake levels are high [5–9].

In 2016, South Africa became the first African country to approve oral daily PrEP and it continued to be the only PrEP modality approved for use in South Africa at the time of this study. UNAIDS 2025 PrEP targets recommend PrEP uptake levels of 50% among MSM and TG communities with incidence rates > 3% [10]. Unfortunately, daily PrEP uptake and continued use have been lower than anticipated [11–13]. Other studies have shown that barriers to PrEP use included taking a daily pill, side-effects from the pill and/or their sexual re-alignment treatment, cost and poor access to quality holistic healthcare [14, 15]. While literature related to the experiences of MSM and TG people in South Africa is limited, previous studies demonstrate varying degrees of PrEP awareness and utilization. One study exploring rates of daily PrEP use among MSM presenting to a health clinic in Cape Town for post-exposure prophylaxis (PEP) initiation found that 90% of MSM participants were aware of daily PrEP, 75% willing to use it, yet only 15% had used it in their lifetime [16]. A study of South African transgender women found that 45% of all participants were aware of daily PrEP, 36% knew where/how to access it, 15% had ever utilized it and 11% were current users [14]. One multi-site study conducted at HIV prevention clinics offering daily PrEP found that 88.5% of MSM participants at the Gauteng and Western Cape sites were aware of PrEP; however, 68.2% had ever used it and 32.9% were current daily PrEP users [17]. In this study, the most frequently reported reasons for MSM discontinuing daily PrEP use were side effects and feeling stigmatized. In another study, Sullivan and colleagues found that when MSM and TG women were offered a comprehensive HIV prevention package (i.e., condoms, lubricant, sexually transmitted infections (STI) screening, couples HIV testing and counseling, PrEP or PEP), daily PrEP uptake was 55.3% [18].

There is a need to better understand what strategies can close the gap in the daily PrEP cascade among sexual and gender minorities in South Africa, especially as new PrEP formulations are being introduced. Therefore, we conducted a mixed-methods study to determine PrEP (oral daily and oral event-driven) utilization, preferences, facilitators, and barriers among MSM and TG people in Soshanguve, Gauteng. Our paper addresses the following objectives: (1) determine utilization rates of oral daily PrEP in this study population, (2) explore attitudes and acceptability of oral daily PrEP, and (3) identify barriers and facilitators to its use. Informed by our findings, we present three strategies to strengthen public health initiatives that respectfully and effectively enhance PrEP uptake efforts among MSM and TG individuals in South Africa. Findings related to event-driven PrEP will be presented in a separate paper.

## Methods

### Study Design

This was a mixed methods study that used a two-phase explanatory sequential design: (1) quantitative analysis of cross-sectional surveys followed by (2) qualitative analysis of in-depth interviews (IDI).

#### Study Setting

This study was conducted in Soshanguve. This township is in the City of Tshwane district, a peri-urban zone in northwest Gauteng, South Africa. In 2019, HIV prevalence in Tshwane was 23.1% [19]. In Soshanguve, oral daily PrEP is available free of cost in the public sector and at non-governmental organizations and is available for a cost in the private sector. Study procedures were led by a clinical research team at the Setshaba Research Centre, a community-based non-governmental research organization located in Soshanguve.

#### Sampling

Inclusion criteria were being at least 18 years of age; self-identifying as a gay man, other MSM, or TG person; residing in Tshwane and being literate in at least English or Setswana. We recruited individuals with personal experience using PrEP and those without to gain insight into the facilitators and barrier to PrEP use. There were no exclusion criteria related to HIV status to ensure a breadth of perspectives related to HIV prevention and navigating HIV risk. People living with HIV are equally affected by, and concerned with, preventing transmission of HIV as those not living with HIV, thus, we did not want to discount their voices and we hypothesized that their perspectives would add insight into additional strategies to expand PrEP use.

We planned to recruit up to 300 survey participants with the aim of enrolling a minimum of 200, based on the information provided by local Lesbian, Gay, Bisexual, Transgender, Queer, Intersex (LGBTQI) organizations on the potential number of participants that could be reached within the recruitment period. For the in-depth interviews (IDIs), we purposively chose a sample of survey participants who were interested in participating in the IDIs based on gender identity, age, and HIV status, and of those that were contactable and available. We conducted interviews until we determined that we had achieved sufficiency based on observations that the perspectives and experiences shared were not yielding new concepts (determined by the investigators conducting interviews and concurrently coding interview transcripts) [20].

### Phase 1: Cross-Sectional Survey

#### Data Collection

We recruited survey participants between July and August 2021 through word-of-mouth, snowball sampling, and outreach efforts at local LGBTQI organizations and publicly-funded health clinics in Soshanguve. A trained community engagement team recruited participants in partnership with two peer recruiters. The peer recruiters were active members in the Soshanguve community; one identified as a gay cis-gendered man and the other as a transgender woman.

The recruitment team provided all potential participants with a study information sheet and the opportunity to ask questions. Interested participants were invited to complete an electronic eligibility screening questionnaire and provide informed consent through a Research Electronic Data Collection (REDCap) survey. If eligibility criteria were not met or informed consent not provided, participants were not directed to the study survey. If eligible and consented, participants were directed to a self-administered REDCap survey, in English or Setswana according to participant preference. The survey took 10 − 20 min to complete and participants received ZAR50 (approximately US$2.68) for their participation.

**Fig. 1:**
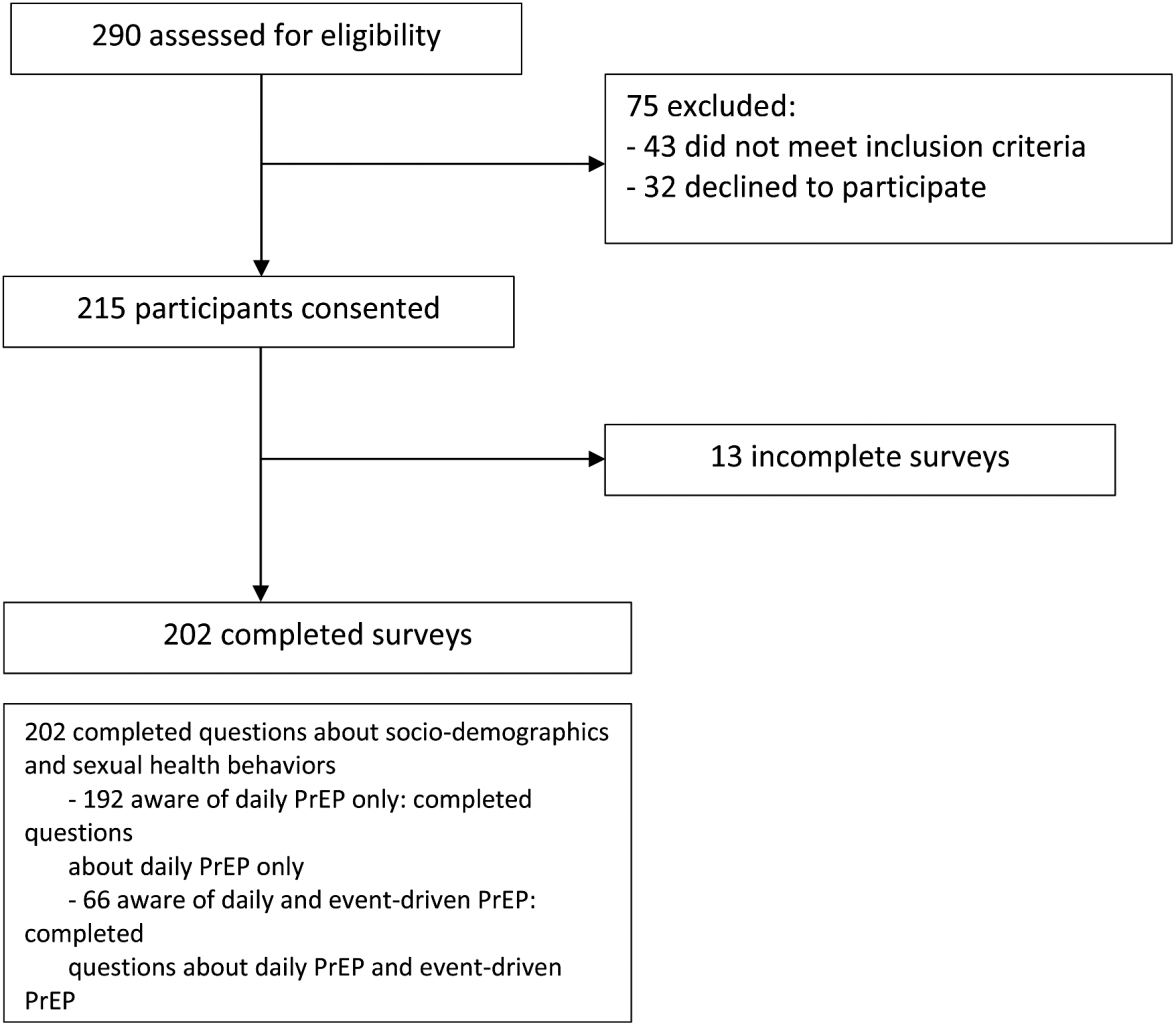
Survey participant enrollment

The survey was developed by the study team. The survey deployed multiple-choice, checkbox or Likert-scale questions; there were no open-ended questions. Part 1 of the survey related to socio-demographic information and sexual health: i.e., income and education levels, gender identity, self-reported HIV status, self-perceived HIV risk status and sexual health behaviors. The survey deployed a branching logic based on participant responses: all participants completed Part 1, those aware of PrEP were additionally directed to complete Part 2 (Fig. 1). Part 2 included questions related to acceptability, utilization, and access to daily PrEP. A modified PrEP Stigma Scale [21] was included to explore attitudes towards PrEP. Part 3 related to acceptability of event-driven PrEP and whether participants have a preference between daily and event-driven PrEP. The focus of this paper is daily PrEP; thus, we will not be presenting data related to Part 3. See Appendix for complete survey.

#### Analysis

Survey data were analyzed using STATA 15 [22]. We used descriptive statistics to present participant sociodemographic information and sexual health behaviors. We assessed associations between the sociodemographic information, sexual health behaviors, and PrEP outcomes using the Chi-square test or Fisher’s exact test, as appropriate. We used univariate logistic regression to analyze variables associated with oral daily PrEP willingness, awareness, and engagement, and reported using odds ratios (ORs) and 95% confidence intervals (CIs). We considered p-values < 0.05 statistically significant.

Regarding sex and gender: we defined transgender women as both (1) reporting male sex at birth and (2) reporting gender identify as “female,” “transgender man” or “transgender woman.” We defined transgender men as both (1) reporting female sex at birth and (2) reporting gender identify as “male,” “transgender man” or “transgender woman.” We defined “men who have sex with men” (MSM) as cis-gender men who reported having sex with “Men (not transgender),” “Men (transgender),” or “Women (transgender)” (to capture those having sex with individuals assigned male sex at birth and individuals identifying with the male gender).

Regarding PrEP awareness and acceptability: Participants were considered PrEP unaware if they answered, “I have never heard of PrEP before” to the question, “What do you know about Pre-Exposure Prophylaxis (PrEP)?” They were considered PrEP aware if they chose any of the alternative answer options: “It is a pill for curing HIV,” “It is a pill for preventing HIV,” or “I have heard of PrEP but I do not know what it is for.” Participants were considered PrEP willing if they agreed with the statement “I believe that PrEP is a suitable HIV prevention method for me.” During the analysis phase, we determined that the survey questions did not completely or directly elicit knowledge of PrEP. Thus, we did not use any of the survey data to analyze PrEP knowledge and instead explored this concept with the IDI data.

### Phase 2: In-Depth Interviews

#### Data Collection

IDI participants were recruited from the survey sample (participants could indicate their interest in participating in IDIs at the end of the survey) and continued recruitment efforts by the community outreach team. As best they could, the recruitment team aimed to achieve a balanced representation between gender (MSM vs. TG) and HIV status (self-reported HIV negative vs. HIV-positive) within the IDI sample; ultimately, the sample was ultimately unbalanced due to challenges with obtaining consent and reaching participants telephonically.

Based on preliminary findings from the cross-sectional survey, a semi-structured guide was developed to further explore the patterns of PrEP use observed in the quantitative data. The IDI guide was piloted with the peer recruiters and it was revised based on their feedback for clarity, understanding, and relevance. Topics included: gender and sexual identity, experience with HIV-related services in Tshwane, the impact HIV has had on their life and sexual behaviors, knowledge of HIV prevention and PrEP, attitudes and preferences towards PrEP modalities and services, and personal experiences with daily PrEP. The IDI guide was written in such a way that both HIV-negative and HIV-positive individuals could engage in a conversation about PrEP. With the understanding that HIV-positive individuals are not eligible to use PrEP but still had valuable perspectives to contribute, we asked these participants to discuss acceptability and preferences related to PrEP in the hypothetical scenario of being HIV-negative (e.g., prior to HIV diagnosis).

Two trained researchers who identify as women conducted IDIs, which each lasted 20 − 60 min and were conducted in English or Setswana. The interviews were conducted in a private office at the research center. Participants were compensated ZAR75 (approximately US$4.04) for completing the IDI. IDIs were audio-recorded with participant permission, professionally transcribed, and translated into English.

#### Analysis

We employed thematic analysis [23] in Dedoose (Version 9.0.46). Two investigators (I.P.U., N.H.) read IDI transcripts for immersion and developed a preliminary codebook based on *a priori* codes from the interview guides and emergent codes. Investigators independently coded one transcript, then came together to discuss and revise the codebook where appropriate. They used the codebook to code the remaining transcripts, resolving discrepancies through discussion (i.e., comparing codes applied by each analyst, explaining rationale for code, ensuring adherence to codebook definitions) to come to inter-coder agreement [24, 25].

A standard matrix was created to display themes, subthemes, and their definitions, and accompanying illustrative quotes. An investigator (J.D.) reviewed the thematic matrix to ensure accurate representation of the data. We also compared the perspectives and experiences between different groups in our sample: those who have never used PrEP vs. those who have (to elicit facilitators and barriers to use), those living with HIV vs. those not (to explore the potential role people living with HIV can play in HIV prevention), and those receiving services from LGBTQI organizations vs. those not (to explore the impact of different health service delivery models).

Since the IDI facilitator’s guide was informed by our preliminary survey findings, we organized and merged the quantitative results with the qualitative themes to identify areas of convergence or divergence. The key themes identified from our quantitative survey were the following: oral daily PrEP uptake, factors influencing daily PrEP awareness, factors influencing daily PrEP willingness, and factors associated with PrEP utilization. We then compared how the qualitative findings either supported or disputed the qualitative findings within the same theme. In our discussion, we offer a critical analysis of possible hypotheses for contradicting findings between the qualitative and quantitative data. Utilizing a thematic approach, we present key qualitative findings that could explain or expand our understanding of the PrEP uptake, awareness, willingness and utilization patterns uncovered in the quantitative study phase.

### Reflexivity

#### Prior Assumptions and Experience

Members of the research team engaged in face-to-face contact with study participants were required to evaluate how their interactions with participants might be influenced by their own professional background, experiences, and prior assumptions in the context of the current study. Both interviewers were social scientists with clinical experience. A key point we wanted to address when drawing conclusions from the data was whether participants’ willingness to communicate openly about their experiences was influenced by our professional background, or how this knowledge influenced what was said. It was crucial to create an atmosphere of openness and build trust with our study participants. Interviewers and study personnel are acutely aware of the privileges and positions they hold as individuals working with in the healthcare system and academia: institutions that have a complicated, and oftentimes fraught, relationship with minoritized communities. This was achieved by working closely with our community engagement team, a team that has invested years building trusting relationships within the community. Further, we invited individuals from the community who identify as sexual and gender minorities to join the community engagement team’s recruitment efforts and reviewed our study documents with them to further evoke their insight and feedback on the questions we hoped to ask our participants. In addition, we prioritized the individual needs of our participants to support their participation in the study: for example, offering transportation to and from interview sites.

### Ethical Considerations

Ethical approval was received from the University of Witwatersrand Human Research Ethics Committee (HREC) (Ethics Reference No. 210,218).

## Results

Survey participants were recruited between July and September 2021. Two hundred and ninety people were screened, of whom 247 (85%) met eligibility criteria, 215 (74%) provided digital consent to participate and 202 (70%) valid surveys were completed (Fig. 1). Surveys that were started but not completed were considered invalid and excluded from analysis. Twenty participants completed IDIs between October 2021 and March 2022.

### Participant Socio-Demographics

Half (51%, *n* = 103) of survey participants were younger than 30 years old (Table 1). The majority self-identified as MSM (70%, *n* = 141), 24% (*n* = 48) as transgender women, 4.5% (*n* = 9) as transgender men and 1.5% (*n* = 3) with another gender (i.e., gender nonconforming/non-binary). Since there was a very small proportion of transgender men (4.5%, *n* = 9), they were combined with transgender women and presented as transgender (28.5%, *n* = 57) in the final analysis. The majority of TG (79%, *n* = 45) reported the current use of gender-affirming hormones (e.g., estrogen, testosterone). One-quarter (24%, *n* = 47) of survey participants self-reported being HIV-positive, 55% (*n* = 109) HIV-negative, and 22% (*n* = 44) were unsure of their HIV status (“HIV unsure”). More than half of the participants (59%, *n* = 118) were unemployed, 63% (*n* = 125) had an average monthly household income < ZAR 5,000^1^ and 36% (*n* = 73) had post-secondary or higher education.

**Table 1:** Survey participant socio-demographics

	Current or past daily PrEP users (n = 30)	All participants (N = 202)
Age		
18–29 years old	21 (70%)	103 (51%)
30–39 years old	9 (30%)	87 (43%)
40 + years old	0 (0%)	12 (6.0%)
Gender identity^1^		
Cisgender man	16 (53%)	141 (70%)
Transgender woman	10 (33%)	48 (24%)
Transgender man	1 (3%)	9 (5%)
Gender non-binary	1 (3%)	2 (1%)
Another term of identity	1 (3%)	1 (0%)
Currently using gender-affirming hormones	9 (30%)	45 (22%)
Unemployed	18 (60%)	118 (59%)
Average monthly household income^2^		
<R5,000	17 (57%)	125 (63%)
R5,001–R10,000	4 (13%)	34 (17%)
R10,001–20,000	8 (27%)	31 (16%)
>R20,000	1 (3%)	7 (4%)
Highest education obtained		
Grade 12 or below	16 (53%)	129 (64%)
Diploma or above	14 (47%)	73 (36%)
Relationship status^1^		
Single, never married	25 (83%)	163 (81%)
Co-habitating	2 (7%)	20 (10%)
Married	0 (0%)	7 (4%)
Separated or Divorced	2 (4%)	8 (7%)
Other	0 (0%)	3 (2%)
HIV status (self-reported)^2^		
HIV-positive	5 (17%)	47 (24%)
HIV-negative	22 (73%)	109 (55%)
Unknown	3 (10%)	44 (22%)

1.Missing 1 data point; 2. Missing 2 data points

Among survey participants who were HIV-negative or -unsure: 59% (*n* = 91) reported their most recent HIV test to be less than 6 months ago, 33% (*n* = 51) more than 6 months ago, and 6% (*n* = 9) had never tested before. Most considered themselves at “low risk” for HIV acquisition 67.5% (*n* = 102), 23.1% (*n* = 35) at “medium risk,” and 9.3% (*n* = 14) at “high risk.” The number of HIV susceptibility factors a participant reported was associated with their self-perceived likelihood level of HIV acquisition [*X*^2^.(2, *N =* 151) = 16.61, *p* < .001): “low risk” participants endorsed an average of 2.0 factors, 2.8 factors for “medium risk,” and 3.9 factors among “high risk.”

Among all survey participants, a majority (89%, *n* = 176) reported engaging in anal sex in the last 6 months. 12% (*n* = 24) reported using condoms “less than half the time” and 10% (*n* = 20) reported “never or rarely” using condoms in the last 6 months. In addition, 25% (*n* = 51) had a lifetime history of at least one STI, and 4% (*n* = 9) had engaged in transactional sex (i.e., exchange sex for money, gifts, or shelter) in the last 6 months.

For the in-depth interviews, data sufficiency was obtained with twenty participants. The twenty IDI participants ranged from 18 to 45 years old. Thirteen identified as cisgender men, five identified as transgender women and two identified as gender non-binary/nonconforming. Eleven IDI participants reported living with HIV.

### Oral Daily PrEP Uptake

**Fig. 2:**
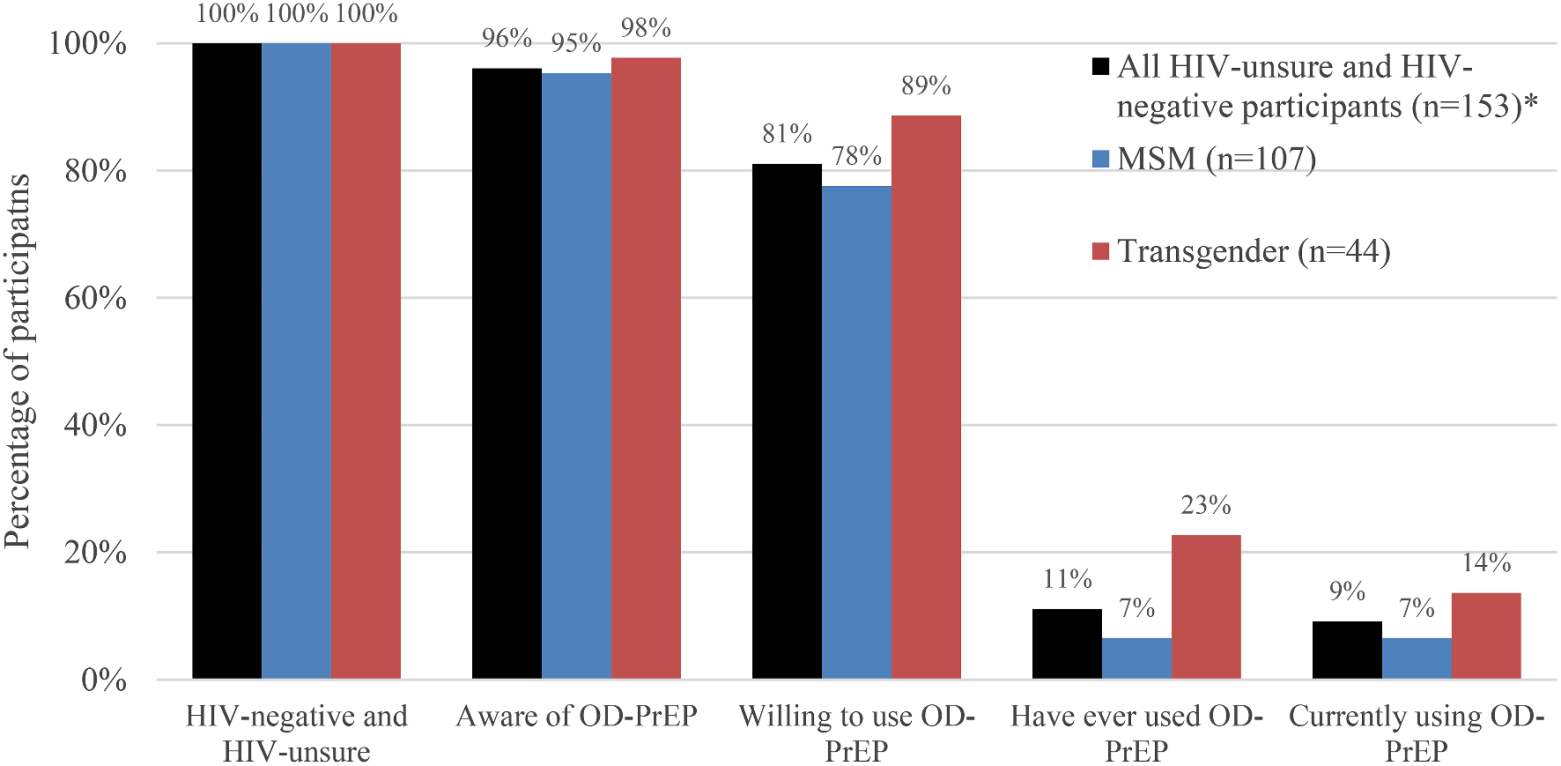
Oral daily PrEP cascade among HIV-negative and HIV-unsure participants. *2 participants not represented in MSM or TG categories given that they reported different identities

95% (*n* = 192) of survey participants had heard of oral daily PrEP before. Of the 192 who were aware: 154 (80%) had never used it, 16 (8%) were currently using it, and 14 (7%) had used it in the past. Among those who were not HIV-positive and had never used PrEP (the target audience for PrEP uptake efforts), 81% (*n* = 124) were willing to use it (Fig. 2).

Of the twenty IDI participants, three were current daily PrEP users and two were former daily PrEP users; the remainder had never used it before. Among the 15 never users, five were HIV-negative and 10 were living with HIV. One of the former daily PrEP users was now living with HIV.

### Factors Influencing Daily PrEP Awareness

#### Surveys

The most frequently reported source of PrEP information was LGBTQI organizations (52%), followed by friends/family (45%) and social media (42%). 81% (*n* = 154) of PrEP-aware survey participants agreed with the statement, “I need more information about PrEP,” including 53% (*n* = 16) of current and former daily PrEP users. Agreeing with this statement was associated with PrEP willingness [*X*^2^.(1, *N =* 144) = 5.52, *p* = .018], whereas disagreeing with this statement was associated with PrEP use [*X*^2^.(1, *N =* 145) = 15.16, *p* < .0001].

#### In-Depth Interviews

All IDI participants correctly identified the purpose of daily PrEP. Some participants provided analogies to explain PrEP, comparing it to contraception, antibiotics, or vaccines. Most daily PrEP-naive IDI participants understood that it is taken daily; however, one participant believed it is dosed monthly and two participants believed that it is meant to be taken only before sex. One former daily PrEP user recounted taking it only before sex, citing that the counselling he received was unclear. Three correctly explained that efficacy decreases with less adherence. Two daily PrEP-naive participants believed that it is only meant to be taken by gay men. Among the 15 participants who were never users, nine could identify at least one place where one could obtain PrEP.

When asked to explain the difference between “pre-exposure prophylaxis” (PrEP), “post-exposure prophylaxis” (PEP), and “antiretroviral therapy” (ART), most participants were unfamiliar with the term “antiretroviral therapy/ART,” even if they were HIV-positive. When the term was explained, participants recognized the concept but not the term: instead, they understood HIV treatment to be “ARVs.” Participants were most unfamiliar with PEP. Four IDI participants stated that, given that PrEP and ART both involve the use of antiretrovirals (ARVs), it is easy for community members to assume that a daily PrEP user is HIV-positive or “sick.”

The way I see it, is that to them it’s the same as taking ARVs, so according to them there is no difference, so the person taking PrEP and the one taking ARVs they are like the same to the community, there is no difference to them.

25–30 year-old transgender woman, HIV-positive, daily PrEP never user

The bottles are in an ARV-like kind of bottle. So, for me, I just put the bottles at home, and they never ask me, but I think they assume I am HIV [positive] because I am taking ARVs.

25–30 year-old MSM, HIV-negative, current daily PrEP user.

The nine IDI participants who had an ongoing relationship with an LGBTQI organization (i.e., receive HIV-related services, participated in their educational events) tended to have more detailed knowledge about daily PrEP and HIV prevention:

What I know about PrEP is that it’s medication that you take when you are HIV-negative to provide you with some sort of immunity for HIV. You take it daily for high protection: the less you take it, the less you are protected. I heard about PrEP at OUT, the organization that I told you about. As soon as I was done with my PEP, they advised me to be on PrEP; and it took 28 days when we initially started where you can be on the safe zone. When I drink it every day, I am 90 plus [percent] protected.

25–30 year-old MSM, HIV-negative, current daily PrEP user.

Five daily PrEP-naive participants cited lack of knowledge as personal reasons for never initiating daily PrEP:

Because I don’t know a lot about it and I am still researching about PrEP, I would want to know the side effects before taking it.… If I can get somebody who can tell me everything—what it means and how it’s started—that’s what I need, then I will take it.

18–25 year-old male, HIV-negative, daily PrEP never user.

Nearly all IDI participants felt that, while awareness was high, community knowledge about PrEP was poor due to lack of education efforts and discourse. Participants identified a lack of “door-to-door” PrEP outreach efforts and workshops at community sites (e.g., schools, clinics, police stations, “gay clubs,” HIV testing centers). Three participants suggested that educational efforts be led by PrEP “guides” who can demystify the process, answer questions, and offer “step-by-step” direction. Two participants specified that educational leaders should be “peers” or identify as LGBTQI.

Both PrEP users and never users described being introduced to daily PrEP by a close friend. Similarly, PrEP users described introducing daily PrEP to their close friends and partners; thus, engaging in informal, yet substantial, peer education about PrEP:

[I tell them,] ‘you know that there is a pill that prevents HIV?’ and everyone is just, like, interested. So, it’s interesting to teach them and advise them on how to go through the procedure and also be on the pill. So, with my friends, also, they know I’m taking PrEP. I’ve taught me about PrEP, so it’s an interesting journey to teach them…. At home, the bottles are all over, I am not hiding them–but it’s not a stigma, it’s not uncomfortable, the conversation is just interesting.

25–30 year old MSM, HIV-negative, current daily PrEP user.

### Factors Influencing Daily PrEP Willingness

#### Surveys

19% (*n* = 25) of PrEP never users, 17% (*n* = 2) of past users and 15% (*n* = 2) or current users did not find PrEP to be a suitable HIV prevention method for them. Age, gender identity, highest education level, and income were not significantly associated with PrEP willingness (Table 2). Participants who received their HIV information from friends and family were more likely to be willing to use PrEP, compared to those who did not receive HIV information from this source [OR = 2.50, 95% (1.04, 5.97); *p* < .05]. Among participants who were HIV-negative/unsure, their self-perceived HIV risk level was not associated with willingness to use PrEP [*X*^2^.(1, *N =* 118) = 0.14, *p* = .711].

**Table 2:** Factors associated with daily PrEP willingness and utilization

		Daily PrEP willingness	Daily PrEP use (current or past)
		OR (95%CI)	OR (95%CI)
**Socio-demographics**			
Age group			
	18–29	1.0	1.0
	30–39	0.79 (0.35, 1.76)	0.47 (0.20, 1.10)
	> 40	1.67 (0.20, 14.05)	- ^†^
Gender identity			
	Cisgender man	1.0	1.0
	Transgender	2.26 (0.82, 6.28)	1.81 (0.78, 4.21)
Highest educational level obtained			
	Grade 12 or below	1.0	1.0
	Diploma or above	0.81 (0.36, 1.80)	1.64 (0.75, 3.60)
Income level			
	<R5,000	1.0	1.0
	R5,001–10,000	0.83 (0.30, 2.29)	0.86 (0.27, 2.75)
	R10,001–20,000	1.73 (0.48, 6.27)	2.09 (0.80, 5.42)
	>R20,000	1.15 (0.13, 10.12)	1.0 (0.11, 8.83)
**Sexual health information**			
Source of HIV information^#^			
	LGBTQI organizations	0.56 (0.23, 1.33)	1.32 (0.58, 3.0)
	Social media	0.75 (0.33, 1.72)	2.01 (0.84, 4.79)
	Friends/family	2.50 (1.04, 5.97) **	1.05 (0.48, 2.30)
Source of PrEP information^#^			
	LGBTQI organizations	0.99 (0.45, 2.20)	1.24 (0.56, 2.75)
	Social media	0.83 (0.38, 1.84)	0.97 (0.44, 2.13)
	Friends/family	2.22 (0.95, 5.18)	0.83 (0.38, 1.83)
HIV risk self-assessment			
	Low risk	1.0	1.0
	Medium or high risk	1.14 (0.41, 3.18)	2.22 (0.92, 5.33)
Have a sexual partner on daily PrEP			
	No	1.0	1.0
	Yes	0.56 (0.22, 1.38)	2.60 (1.09, 6.18) **
Has a close friend or family member on daily PrEP			
	No	1.0	1.0
	Yes	1.56 (0.69, 3.50)	1.54 (0.70, 3.37)

**p < .05

#reference category are those that answered in the negative

†No participants in this subset were 40 years or older

Table 3 presents survey findings related to attitudes around PrEP access, stigma, and its impact on health. A minority of HIV-negative/unsure participants agreed with stigmatizing statements related to hiding their PrEP use from others (16%, *n* = 24) and fearing repercussions at work for using PrEP (7%, *n* = 10). A majority endorsed comfort with discussing PrEP with their partners (82%, *n* = 119) and this was significantly associated with PrEP willingness [*X*^2^(1, *N* = 143) = 8.82, *p* = .003]. 36% (*n* = 52) of HIV-negative/unsure participants agreed with “If I were to use PrEP, people would think that I have sex with a lot of people,” compared to 60% (*n* = 15) of those with experience using daily PrEP. Agreeing with this statement was significantly associated with daily PrEP utilization [*X*^2^.(1, *N =* 145) = 11.15, *p* < .001] but not PrEP willingness [*X*^2^.(1, *N =* 143) = 1.15, *p* = .283].

**Table 3:** Attitudes associated with PrEP willingness and use among 146^1^ HIV-negative and HIV-unsure participants

Agree with the following statements (n, %)	Daily PrEP willingness (*n* = 124)	Daily PrEP use (*n* = 25)	All HIV-negative and -unsure participants (*n* = 146)
**Access to PrEP**			
“I need more information about PrEP”	106 (85%) **	14 (56%)^†^ **	119 (82%)
“It is difficult for me to access PrEP.”	42 (34%)	7 (28%)	46 (32%)
“I would only use PrEP if it was free of cost.”	51 (41%)	12 (48%)	57 (39%)
“The way PrEP is used, and how often it must be used, it a barrier for me.”	36 (24%)	9 (36%)	40 (27%)
“There are easier ways to keep from getting HIV than taking PrEP.”	69 (56%)^†^	10 (40%)^†^	79 (54%)
**PrEP stigma and disclosure**			
“If my employer found out that I was using PrEP, I might lose my job”	9 (6%)	2 (8%)	10 (7%)
“If I were going to use PrEP, I would feel a need to hide that from other people.”	21 (15%)	6 (24%)	24 (16%)
“I would feel comfortable talking to my partner about using PrEP.”	99 (80%)^†^ **	20 (80%)^†^	119 (82%)
“If I were to use PrEP, people would think that I have sex with a lot of people.”	37 (30%)^†^	15 (60%)^†^ **	52 (36%)
**Impact of PrEP**			
“I’m concerned about the side effects of PrEP.”	68 (55%)	12 (48%)	80 (55%)
“I’m concerned that PrEP may interact with my hormones.”	53 (43%)^†^	8 (32%)^†^	61 (42%)
“I’m concerned that my sex partner(s) will not want to use condoms if I’m on PrEP.”	43 (35%)^†^	12 (48%)^†^	55 (38%)
“I am at enough risk of HIV that I would benefit from PrEP”	93 (75%) **	19 (76%)	101 (69%)

^1^Only participants who indicated being aware of PrEP and self-reported their HIV status to be HIV-negative or unknown

**p < .05

†Denominators in this column are less than 146 because of missing data

27% (*n* = 40) of HIV-negative/unsure participants agreed that the daily PrEP route of administration was a barrier, 32% (*n* = 46) found it difficult to access, 39% (*n* = 57) would only use it at no cost, and 54% (*n* = 79) believed there are easier ways to prevent HIV. A considerable proportion had concerns over PrEP side effects (55%, *n* = 80), possible interactions with hormones 42% (*n* = 61) and sex partner(s) not wanting to use condoms if on PrEP (38%, *n* = 55).

#### In-Depth Interviews

All but one IDI participants were willing to use PrEP and had favorable attitudes towards it. The most cited reason for being willing to use PrEP was the sense of safety and security that it provides, particularly for circumstances related to non-monogamy and sexual assault. The one PrEP unwilling participant felt protected enough with the HIV prevention methods he was deploying and, thus, never felt in need of PrEP. Most of the participants living with HIV were diagnosed prior to the introduction of PrEP; yet all were strong proponents of PrEP and wished it had been available to them before they had seroconverted.

All PrEP never users believed that it would be easy to discuss daily PrEP with their partners; and many expressed an eagerness to start daily PrEP so that they could teach their partners about it:

It would be very easy because we don’t stay in the same place. So, I can tell him that life happens, and mistakes happen, so it’s easier that he takes PrEP and I also take it on the side—to be on the safe side. ‘Cause when he goes to Mpumalanga, and I am in Pretoria, who he meets up with? So, if he drinks it that side and I drink it this side it is safe; even when we meet up, we are both fine.

30–35 year-old MSM, HIV-negative, daily PrEP never user.

Participants cited community perceptions towards PrEP that may be impacting PrEP willingness: its lack of safety, side effect profile, and stigma (consistent with survey findings). Many IDI participants, especially those with experience using PrEP, describe how PrEP use is often associated with promiscuity and sexual irresponsibility:

The most [common] misconception is if you are taking PrEP then you a hoe (laughs), you are promiscuous and, not only that, but you cannot control your urges and are a hyper person…Yah, it means you just want to take the pill so that you can have unplanned sex anywhere, that you cannot control your urges…So it can put you at risk of being hypersexualized and many gay men are hypersexualized…You taking the blue pill proves that, ‘oh, it’s true, these people want to have sex everywhere.’ So that is the thing I see in the community when taking PrEP.

25–30 year-old MSM, HIV-positive, daily PrEP never user.

The following participant describes how this perception was dispelled by the positive messaging he came across on social media:

I follow these guys on twitter who promote PrEP and they really say positive stuff. They show PrEP and show themselves taking PrEP and destigmatizing the whole PrEP intake you know. They have really improved perceptions about taking PrEP. Normally there would be, like, stigma like, ‘Oh my God, we are quite promiscuous, and you are intentionally sexually on a high drive and prone to having sex a lot.’

25–30 year-old MSM, HIV-negative, daily PrEP never user.

In addition, there were reports of discouraging messaging circulating within the community around the safety of PrEP due to the misconception that taking ARVs while HIV-negative will threaten protection from HIV acquisition:

Most of the women that I normally chill out with, they would say, ‘I do not want to touch PrEP,’ because PrEP, ‘when you use it, when you stop, you will be HIV positive.’ Some would say you contract it without being sexually active.

30–35 year-old transgender woman, HIV-positive, daily PrEP never user.

Two daily PrEP never users described the community perception that daily PrEP has burdensome side effects. Four of the five IDI participants who have used daily PrEP endorsed experiencing side effects with PrEP initiation, all of whom characterized the side effects as temporary and manageable (mostly resolving within the first week). For two participants, the sense of protection provided by PrEP afforded them a more tolerable attitude towards the side effects:

For the first seven days I had certain side effects, and these side effects were: being dizzy, fatigue, diarrhea, nausea, and vomiting. But as time went, everything went back to normal, and I was fine. But if I skip maybe two days then some of the side effects come back. But it also makes me feel good and safe because I feel protected.

18–25 year-old transgender/gender non-conforming, HIV-negative, current daily PrEP user.

One former PrEP user discontinued on the second day of use due to side effects; however, during their second attempt they realized the temporality of the side effects and felt more capable and prepared to maintain adherence.

### Factors Associated with PrEP Utilization

#### Surveys

Age, highest education level, gender identity and self-perceived HIV risk was not associated with daily PrEP use (Table 2). Among PrEP never users who were HIV-negative/unsure and willing to use PrEP, 34% (*n* = 35) agreed with the statement “It is difficult for me to access PrEP;” this is in comparison to 28% (*n* = 7) of those with experience using daily PrEP agreeing with this statement (Table 3). 80% (*n* = 24) of PrEP users were/had accessed PrEP from a public health facility; 13% (*n* = 4) from a private health clinic; and 6% (*n* = 2) from an LGBTQI service organization.

Survey participants with more than one close social tie (i.e., sexual partner, friend or family member) on daily PrEP were 2.7-fold more likely to have used daily PrEP themselves [OR = 2.71, 95% CI = (1.05, 6.99), p-value = 0.039], especially if the social tie was a sexual partner [OR = 2.60, 95% CI = 1.09, 6.18), *p* = .027].

Agreeing with the following statement was associated with PrEP use: “If I were to use PrEP, people would think I am having sex with a lot of people” [*X*^2^.(1, *N =* 145) = 11.15, *p* < .001]. PrEP-engaged participants were twice as likely to agree with this statement, compared to HIV-negative/unsure participants who have never used PrEP but were willing to (53% v. 24%, respectively).

#### In-Depth Interviews

Consistent with survey findings, close social ties—sexual partners in particular—played an influential role on PrEP engagement within their social network. IDI participants living with HIV showed a vigilant concern for sexual safety that contributed to an enthusiasm around PrEP. Many engaged in informal and formal efforts to advocate for PrEP and increase awareness among their friends or partners, supporting them in seeking out and navigating PrEP services. This participant described learning about PrEP for the first time and seeing it as an opportunity to disclose his HIV status to his partner, since he felt like PrEP was a tool, he could offer to his partner to reassure him:

We dated for two years before I could tell him that I am HIV positive—and we had always used protection. So, he started wanting us to, uh—because we have been dating for so long, he wanted to stop using protection. And I made a decision that I’m not going to do that if I am not sure where we are as a couple. Then I started hearing about PrEP. After hearing about PrEP, I was like, ‘okay, cool, that’s a good idea. Let me try this: I’m going to use it as an angle for him to understand that we can still live a healthy lifestyle as a couple by doing that.’

35–40 year-old MSM, HIV-positive, daily PrEP never user.

Of the five IDI participants with experience using daily PrEP: four were accessing daily PrEP from an LGBTQI organization and one from a public clinic (in contrast to the survey participants, a majority of which obtained PrEP from public clinics). Eleven IDI participants had ongoing relationships with an LGBTQI organization to access either PrEP, HIV treatment, or education.

LGBTQI organizations were characterized positively and played important roles in their experiences accessing PrEP. These organizations modeled accessible services that met people where they were: e.g., delivering medications to their home, accommodating missed appointments, and regularly checking in on their clients. Services were free and comprehensive: offering full STI panels instead of targeted testing based on symptom screening; encouraging regular HIV testing; and distributing condoms, lubricant, and HIV self-test kits. Participants felt cared for and respected at these organizations. They were seen as confidential and as safe spaces for people of different sexual and gender identities. These organizations also provided educational programs related to HIV-prevention and also LGBTQI identity and rights.

I went to varsity 2015. Then, my sexual life was reckless. Then 2016, I went for my first HIV test with them [LGBTQI organization]: very welcoming, very friendly—took me step-by-step—the counselling was the best. Then it turned out that I am negative in 2016. Then in 2017, I had a reckless sexual encounter and then I went to them. They were very fast; they provided me with the PEP pills, no questions asked. They just had to make sure that I am not exposed to HIV. They provided me with lubes and condoms and pamphlets—like a lot of information. Then around August the same year, in 2017, I started to be on PrEP with them. Ever since, I have been with them. No hesitations. I have missed my appointments, but they have made sure that I do come whenever I am free. I have a good relation with them.

25–30 year-old MSM, HIV-negative, current daily PrEP user.

The two former daily PrEP users had received PrEP services from LGBTQI organizations and discontinued use after the first month because they could not afford to travel to the organization to pick up their medication refill. This demonstrates the importance of delivery services as well as the challenges to providing daily PrEP services, even with well-resourced and accommodating service delivery models.

On the contrary, public clinics were described negatively, with participants characterizing them as inefficient, judgmental, and lacking privacy. Ten IDI participants described the clinics as inefficient, with long waiting times due to lack of personnel and poor management. Ten IDI participants also felt that the clinic staff was discriminatory and judgmental, particularly towards sexual and gender minorities:

At the clinics they will judge you, because when you are at the clinic they will stare, ‘Yoo…’ People will look around and look at you if you are a transgender woman or a gay; and then they will say, ‘this person is gay, and he is wearing a weave.’

18–25 year-old MSM, HIV-positive, daily PrEP never user.

Four IDI participants disliked the lack of privacy at the public clinics, describing a triage system that creates zones for different types of service needs at the clinic. As a result, clients who are directed to a zone are publicly identified with that problem:

Clinics, they have this thing of categorizing, and it is the one that is making it difficult for people to go and take medication. Because they know that class is here to fetch ARVs, so people do not want to go there.

30–35 year-old transgender woman, HIV-positive, daily PrEP never user.

Despite the above challenges, eight IDI participants praised the clinic staff, explaining that most are well-intentioned, caring, and supportive, particularly when patients are in crisis or very sick.

## Discussion

Our findings demonstrate that a significant gap exists in the daily PrEP uptake cascade for MSM and TG people in South Africa, which is consistent with previous studies [4, 5]. Low oral daily PrEP utilization within these communities is not due to lack of interest, but to poor PrEP education efforts, discouraging community-level messaging about PrEP, and lack of PrEP service delivery models that are accessible and culturally respectful of sexual and gender minorities. Drawing upon the experiences, attitudes, and preferences of our study participants, we have identified three public health strategies that can be deployed in the pursuit of closing the daily PrEP uptake gap in these key populations.

### Strategy 1: Demystify Daily PrEP by Deploying Community-Engaged Education Campaigns

The need for robust PrEP education was a through-line in our findings. Increased PrEP education has the potential to resolve personal hesitations towards initiating PrEP, dispel common misconceptions, increase the reach and effectiveness of PrEP services, and normalize its use. An important tenet of such educational efforts should involve reframing PrEP as a healthy, safe, and sexually responsible pursuit. Reports of PrEP’s association with promiscuity and HIV stigma is further compounded by the stigmatization of the LGBTQI identity. This results in the misconception that people who use PrEP are sexually irresponsible when, in fact, utilizing PrEP is a profound display of duty, maturity, and precaution.

Our participants are interested in educational campaigns that deploy street-based outreach, community health workers, and involve multiple sectors of society. The most cited source of PrEP information among survey participants was LGBTQI organizations. Similarly, IDI participants with ongoing relationships with these organizations were more educated on daily PrEP; thus, educational campaigns should certainly involve, or be led by, such organizations.

To further address HIV stigma associated with daily PrEP, educational efforts can explain the multiple uses of ARVs (i.e., PrEP, PEP and ART) and reframe ARVs as a medication that is not solely for “sick” individuals. Many participants understood “ARV” to be synonymous with the treatment of HIV, and this understanding could be contributing to reported community perceptions that PrEP is not safe for HIV-negative individuals. Educational campaigns can provide important clarity and differentiation: given advances in HIV prevention science, ARVs can now be used for both HIV prophylaxis by HIV-negative individuals (e.g., PrEP and PEP) and treatment by HIV-positive individuals (e.g., ART).

Of note, despite showing a strong understanding of risk behaviors for HIV acquisition during the IDIs, we observed both high rates of OD-PrEP willingness and high rates of HIV-negative and HIV-unsure participants self-identifying as “low risk” for HIV acquisition. In addition, self-perceived HIV risk was not associated with being PrEP-willing. This suggests that having a low HIV risk-perception is not inherently a barrier to PrEP engagement. Past literature has found that feeling at enough risk for HIV is a prerequisite motivator for initiating and continuing PrEP use. In contrast to ART, which must be taken for life and is the sole therapeutic option for persons living with HIV, PrEP is intended to be used just during ‘seasons of risk’ and is one of numerous HIV preventive approaches available. As a result, higher discontinuation rates for PrEP treatments can be expected as compared to ART [28, 29]. However, this is not necessarily in conflict with our results. It is possible that our study population operationalizes HIV “risk” differently than those in academia, places a different weight on risk level when engaging in decision-making about PrEP, or holds the perspective that the identity of being MSM or TG in South Africa constitutes a risk factor on its own.

Many PrEP rollout efforts across the globe have focused on populations thought to exhibit “high-risk” HIV behaviors. For example, South Africa deployed a four-stage approach for its PrEP rollout: it was first introduced to female sex workers, followed by people reporting MSM behavior, university students, and then young people. It has been postulated that this staged approach inadvertently stigmatized, or contributed to the stigmatization of daily PrEP, by initially introducing it to highly stigmatized populations [30]. Actions that can be taken by the public health community to shift the focus away from PrEP stigma and mitigate its negative effects include changing the narrative that PrEP is “for people at very high risk for infection” to PrEP guidelines/messaging that emphasize that PrEP is “for people who want to reduce their anxiety about HIV infection and take greater responsibility for their sexual health” [31]. We suggest that public health efforts further complicate what it means to target “high-risk” populations in their PrEP expansion efforts.

Side effects related to daily PrEP use was a concern among participants who have never used PrEP and has been cited in previous studies to be a common reason for PrEP discontinuation [17, 32]. Headache, weight loss, and gastrointestinal symptoms (e.g., nausea, abdominal pain, diarrhea) with daily PrEP initiation is known as “start-up syndrome” [33]. It generally only affects 10% of users and self-resolves within 3 months [34]. While these side effects can affect one’s daily life, there are coping strategies available: e.g., taking PrEP medication with food to reduce side effects, taking the medication at a different time of day, taking complementary medication to treat associated side effects (e.g., antiemetics). A significant portion of our survey participants expressed a concern that PrEP may interact with gender-affirming hormones. Thus, there is benefit in incorporating reassurance and evidence around the safety of concurrently using daily PrEP with feminizing and masculinizing hormone therapy. Educational efforts can equip community members and healthcare providers with information on what to expect when initiating PrEP and how to manage side effects, allowing people to feel more prepared and less apprehensive about PrEP.

### Strategy 2: Capitalize on Existing Peer Networks

Our findings suggest that, in the context of deficient community-wide educational forums or social marketing campaigns, motivated and curious participants turn to peers and social media for PrEP information. Our findings illuminate the role that social networks play in the exchange of PrEP-related information, both negative and positive. Christakis (2020) notes that the health behaviors of one person influences the health behaviors of those within three degrees of separation (i.e., that person’s friend, their friend’s friend, and their friend’s friend’s friend) [35].

While misinformation was largely being exchanged at the community-level about PrEP, it was revealed that reliable and supportive information was being exchanged at the interpersonal level. Daily PrEP users, in particular, are actively engaging their social network in informal PrEP education. In addition, individuals who are living with HIV are strong proponents of daily PrEP and are uniquely positioned to introduce PrEP to people who may not have otherwise sought it out. Individuals living with HIV have personal experiences navigating shame, stigma and perceptions of promiscuity that can be of value to those initiating PrEP. In addition, they have practice with taking a prescription daily and conversations of disclosure. Several studies among MSM demonstrated the desire for peer-matching based on identity considerations and lived experience to access PrEP, and that peer leaders with greater PrEP intentions and those living with HIV were more active in forming new friendships [36, 37]. Thus, there is benefit to involving interested HIV-positive individuals in PrEP educational efforts and developing curricula specific to initiating conversation about PrEP with a sexual partner. We suggest capitalizing on current daily PrEP users and HIV-positive partners as formal peer educators in the pursuit of advancing PrEP uptake.

For many individuals, social media is an additional communal space where individuals can interact with peers that share their identities. Some IDI participants described the important role that social media accounts had in obtaining information about PrEP and destigmatizing its use, demonstrating the utility of social media in PrEP educational efforts. A study in the United States among black males from sexual minorities showed that they preferred having more information about PrEP communicated on social media platforms (e.g., Facebook, Instagram, Pornhub, Snapchat) and hook-up apps to enhance adoption and promote adherence among those already on PrEP and participants saw visibility as a way to support some aspects of normalizing the greater discussion of PrEP [38].

### Strategy 3: Expand Accessible and Culturally Responsive PrEP Service Delivery Models

Participants valued service delivery models that are convenient, accommodating, confidential, free, culturally-responsive and non-judgmental. To IDI participants, LGBTQI organizations were well-resourced and represented these values; however, public clinics did not, and some participants actively avoided them. When asked for suggestions on how to expand PrEP use, participants suggested either better equipping public clinics (e.g., increasing capacity, educate providers, mandate all public clinics distribute PrEP) or, alternatively, decentralizing PrEP services (distributing it only at community organizations and pop-up centers). According to one study conducted in the US in HIV clinics, culturally competent primary care clinicians in HIV care settings provided more equitable care and had better patient outcomes than other doctors who showed lower self-reported cultural competence [39].

LGBTQI organizations were reported to offer a wealth of comprehensive HIV-related services including educational programming in a culturally-responsive manner. These organizations tended to take on a harm-reduction approach to care delivery that allowed them to “meet participants where they are at” (i.e., delivering medications, being non-judgmental, proactive prevention efforts) which circumnavigated systemic barriers to accessing HIV-related services. The LGBTQI organizations and the public clinics also seemed to work independently, with no collaboration or integration of efforts and resources.

### Limitations

One limitation of this study was in our operationalization of sexual and gender identities of our participants. The ways in which sex and gender is operationalized in Western academic spheres do not always translate to the communities we serve. In our analysis, we identified potential confusion around the transgender identity. Academic convention may interpret “transgender man” as a person assigned female at birth whose gender identity is man/male. However, some participants interpreted “transgender man” as a person assigned male at birth who identifies as transgender. As a result, in defining “men who have sex with men” we made the decision to operationalize it as cis-gender men who are having sex with anyone who does not identify as cis-gender woman, since it was unclear how respondents were defining “transgender men” and “transgender women.” Further research that illuminates the sexual and gender identification systems used by our study population would be insightful in better connecting with these communities and informing the methodology of future research.

This study is also limited by our ability to capture and operationalize PrEP knowledge among our participants. Identifying concrete PrEP knowledge gaps would have been helpful insight into participant perspectives about the need for increased PrEP knowledge. Additionally, while snowball sampling was a particularly effective recruitment tool, it may have introduced sampling bias. Furthermore, several findings, such as awareness of PrEP, could have been inflated due to us recruiting from PrEP rollout facilities and LGBTQI organizations. It is likely that these factors/indicators will be lower among people in these key populations that do not access or are linked to such facilities or organizations. Unfortunately, we had an underrepresentation of participants accessing PrEP from public facilities in our IDI sample compared to our survey sample; thus, we lack perspectives related to the specific experience of receiving PrEP at the public clinics. The perspectives we obtained relate to participants’ experiences receiving HIV treatment or general health services from the public clinics. The lack of IDI participants who identified as transgender men due to their inability to be contacted or unavailability was also a limitation. Other limitations include relying on self-reported HIV status without confirmatory testing. Even with these limitations, the convergence of the quantitative and qualitative data allowed for deeper insight into participant perspectives and behaviors. Lastly, the self-administered survey design minimized the potential for social desirability bias.

## Conclusion

Effective PrEP rollout efforts require interventions at the individual, community, and institutional levels. It is possible that strategies deployed during the protracted pursuit of closing the HIV treatment gap can be applied to closing the gap in daily PrEP uptake. Rollout efforts should not be a one-size-fits-all approach; it should prioritize ongoing community engagement and surveying of preferences, particularly as new formulations and methods of PrEP delivery receive regulatory approval/become available for public use. Alongside the existing literature, our findings indicate that oral daily PrEP awareness, willingness and engagement can vary widely across identity groups and social networks, whether or not sexual partners were PrEP users, and with different types of organizations that provide PrEP. Future research is needed to understand the impact of specific educational campaigns on PrEP uptake, and to evaluate what curriculum delivery models are most impactful.

Our findings contribute recommendations for meeting the unique needs and preferences of persons with sexual and gender minority identities in the effort to encourage increased PrEP uptake in South Africa.

### Electronic Supplementary Material

Below is the link to the electronic supplementary material.

Supplementary Material 1

## Data Availability

Upon acceptance, the data underlying the findings of this manuscript used to generate the results will be made available upon request from the CEO of Setshaba Research Centre, Dr Khatija Ahmed at KAhmed@setshaba.org.za.

## Footnotes

1 In 2021, the national poverty line in South Africa was < R1,335 per person per month with an average household size of 3.34 persons [26, 27].
