# Supplementary material for: Strategies to Close the PrEP Uptake Gap Among Transgender People and Men Who Have Sex with Men in Tshwane, South Africa: Perspectives from the Community
Source: AIDS Behav. 2024 Mar 1;28(6):1999–2014. doi: 10.1007/s10461-024-04300-7 (PMC11161427; doi:10.1007/s10461-024-04300-7)
Supplement: Supplementary file 1 — Supplementary Material 1 [file 10461_2024_4300_MOESM1_ESM.docx]

**Strategies to close the PrEP uptake gap among transgender people and men who have sex with men in Tshwane, South Africa: perspectives from the community**

India Perez-Urbano^1*^**,** Athmanundh Dilraj^2^, Annah Pitsi^2^, Naomi Hlongwane^2^, Nada Abdelatif^3^, Janan Dietrich^4,5^, Khatija Ahmed^2,6^

**SUPPLEMENTARY APPENDIX**

**Cross-sectional Survey Questionnaire**

Socio-demographics

1. What is your age?

1. 18 – 19 years
2. 20 – 29 years
3. 30 – 39 years
4. 40 – 49 years
5. 50 – 59 years
6. 60 years or older

2. What is your sex at birth?

1. Male
2. Female

3. What is your gender identity?

1. Male
2. Female
3. Transgender man
4. Transgender woman
5. Prefer to self-identify (please specify): ­­­­________

4. What is your race?

1. Black/African
2. White
3. Asian
4. Indian
5. Coloured
6. Other (please specify): ________________

5. Are you employed?

1. Yes
2. No

6. What is your average household monthly income (total average monthly income of everyone in the household)?

1. Less than R5000
2. R5001─10000
3. R10 001–20 000
4. More than R20 000

7. How would you describe your relationship status at the moment?

1. Single, never married
2. Married
3. Divorced
4. Widowed
5. Separated
6. Co-habitating (living together with a partner)
7. Other (please specify): ___________

8. What is the highest education that you obtained?

1. Below grade 10
2. Grade 10─12
3. Diploma
4. Graduate
5. Post-graduate

HIV Information

1. How do you normally get information about HIV? (Select all that apply.)

- 1. Television and/or radio
  2. Social media (e.g., Facebook, Instagram, Twitter, etc.)
  3. Friends/Family
  4. Relationship/sexual partners
  5. A healthcare professional (e.g., doctor, nurse, pharmacist)
  6. An HIV or LGBTQ service organization
  7. Newspaper and/or magazines
  8. Internet websites
  9. None of the above
  10. Other (please specify): __________

2. What is your HIV status?

1. HIV positive [Skip to question #6]
2. HIV negative
3. I don’t know

3. When was your most recent HIV test done?

1. I have never tested for HIV
2. Less than 6 months ago
3. More than 6 months

4. How at risk do you think you are for getting infected with HIV?

1. Low risk
2. Medium risk
3. High risk

5. What reasons contribute to this assessment of your HIV risk level? (Select all that apply based on your practices in the past 6 months.)

1. I don’t use condoms consistently or at all
2. I have an HIV-positive sexual partner who is not on treatment
3. I have unprotected sex with multiple partners (2 or more)
4. I participate in anal sex
5. I exchange sex for money, gifts or shelter
6. I engage in sexual activity while under the influence of alcohol or drugs
7. Other (please specify): ___________

6. Who are you having sex with? (Select all that apply.)

1. Men (not transgender)
2. Men (transgender)
3. Women (not transgender)
4. Women (transgender)
5. People of other genders, not listed above

7. How many sexual partners have you had in the past 6 months? ________

8. Have you had anal sex in the past 6 months?

1. Yes
2. No

9. During the past 6 months, how often have you used condoms during sex?

1. Never or rarely
2. Less than half of the time
3. Most of the time
4. Always

10. Have you EVER been told by a healthcare provider that you have a sexually transmitted infection (STI)? For example: Chlamydia, Gonorrhoea, Syphilis, Genital Herpes, Hepatitis B virus or Human Papillomavirus (HPV).

1. Yes
2. No [Skip to question #12]

11. How many sexually transmitted infection(s) (STI) have you had in your lifetime? ______

12. Are you circumcised?

1. Yes
2. No
3. Not applicable

13. Are you currently taking any hormones or gender-affirming medications (e.g. estrogen, testosterone, spironolactone)?

1. Yes
2. No

14. What do you know about Pre-exposure Prophylaxis (PrEP)?

1. It is a pill for curing HIV.
2. It is a pill for preventing HIV.
3. I have heard of PrEP but I do not know what it is for.
4. I have never heard of PrEP before. [Skip to end]

PrEP Knowledge, Access and Engagement

1. How did you first find out about PrEP? (Select all that apply.)

1. Television and/or radio
2. Social media (e.g., Facebook, Instagram, Twitter, etc.)
3. Friends/Family
4. Relationship/sexual partners
5. A healthcare professional (e.g., doctor, nurse, pharmacist)
6. An HIV or LGBTQ service organization
7. Newspaper and/or magazines
8. Internet websites
9. None of the above
10. Other: (please specify) __________

2. Who should use PrEP?

1. Anyone who wants it
2. Only people at high risk for HIV
3. Only discordant couples (that is, when one person is HIV positive and the partner is HIV negative)
4. Other (please specify): _____________

4. True or false: People should use condoms in addition to PrEP.

1. True
2. False

5. True or false: PrEP can be used by HIV-infected individuals.

1. True
2. False

6. Do you know of anyone who is using, or has used, PrEP?

1. Yes, my sexual partner(s)
2. Yes, a close friend or family member
3. Yes, an acquaintance or someone in my social circle
4. No, I don’t know of anyone who has used PrEP

7. Do you have any personal experience using PrEP?

1. Yes, I am currently using PrEP
2. Yes, I have used it in the past but I stopped using it
3. No, I tried to get PrEP and couldn’t access it [Skip to Part F]
4. No, I have never used PrEP [Skip to Part F]

8. Where/How do you usually access PrEP in your area?

1. Privately over the counter
2. Buy online
3. Public healthcare facility
4. Other (please specify): _________

9. How easy is it to access PrEP in your area?

1. Very easy
2. Somewhat easy
3. Somewhat difficult
4. Very difficult

10. How do/did you usually use PrEP?

1. Daily
2. Only when I engage in sexual activity, no matter the risk
3. Only when I think I am going to engage in high-risk sexual activity
4. Only after I think I may have been exposed to HIV
5. Other way: (please specify): ________________

PrEP Decision Making

Please tell us if you “agree” or “disagree” with the following statements:

1. “I am at enough risk of HIV that I would benefit from PrEP.”

2. “I need more information about PrEP.”

3. “If my employer found out that I was using PrEP, I might lose my job.”

4. “If I were going to use PrEP, I would feel a need to hide that from other people.”

5. “It is difficult for me to access PrEP.” (For example: It is not covered by my insurance, I can’t afford it, I can’t access a distribution site., etc.)

6. “I would only use PrEP if it was free of cost.”

7. “The way PrEP is used, and how often it must be used, is a barrier for me.”

8. “I’m concerned about the side effects of PrEP.”

9. “I’m concerned that PrEP may interact with my hormones.”

10. “There are easier ways to keep from getting HIV than taking PrEP.”

11. “I’m concerned that my sex partner(s) will not want to use condoms if I’m on PrEP.”

12. “I would feel comfortable talking to my partner about using PrEP.”

13. “If I were to use PrEP, people would think that I have sex with a lot of people.”

14. “I believe that PrEP is a suitable HIV prevention method for me.”

Knowledge and Acceptability of event-driven PrEP

1. Have you heard about on-demand PrEP? (Also called “event-driven PrEP” or “2-1-1 PrEP”).

1. Yes
2. No [Skip to end]

2. With on-demand PrEP, the pills are taken:

1. Before sex only
2. After sex only
3. Before and after sex
4. I don’t know

3. Would on-demand PrEP be an acceptable method for you to prevent HIV?

1. Yes
2. No

4. Which PrEP method would you prefer most?

1. On-demand PrEP
2. Daily PrEP
3. I don’t have a preference
4. I am not sure

**In-depth Interview Guide**

Demographics:

1. To start, can you tell me a little bit about yourself?
2. How would you describe yourself in terms of gender?
   1. *We define gender as that you, in your head, think about yourself: cis-gendered man, cis-gendered woman, transgender man, transgendered woman. Cis gender refers to identifying with the same gender as that assigned at birth, whereas transgender is identifying your gender different from the sex assigned at birth, e.g., more masculine/manly, more feminine/womanly, in the middle or none of these.*
   2. When you were born, what was your sex: male or female?
3. How would you describe your sexual orientation? So, what types of genders do you find yourself attracted to and/or having sex with? Men, women, transgender people, nonbinary people, etc.
   1. *We define this as who you are physically, emotionally and spiritually attracted to, based on their sex/gender in relation to your own: straight, gay, bisexual, pansexual.*
4. What are some ways that you would identify/classify yourself among your peers and in your community?
   1. Can you list some categories or labels that you (and your community) use to describe a person’s gender identity or sexual orientation?

Experience with HIV:

1. Please tell me what you know about HIV.
2. What do you think about going to clinics/hospitals in Gauteng?
   1. What do you like/dislike?
3. How has HIV, or the risk of getting HIV, affected your own life?
   1. Note: Ask about HIV status if not brought up naturally.
4. Are you involved with, or receive HIV services from, any local LGBTQ organizations? If so, tell me about how they have contributed to your life?
   1. What do you like about that organization?
5. How do you feel the COVID-19 pandemic has affected your ability to access HIV-related services?

Personal HIV Risk and Prevention Needs Assessment:

1. On a scale of 1 to 5, how much at risk of HIV infection do you consider yourself? With 1 being lowest risk and 5 being highest risk? And why? *(e.g., in a relationship with a person living with HIV, inconsistent condom use, sharing syringes/injection equipment, exchanging sex for money or goods)*
   1. Follow up with participant: asking what would make them a lower or higher number. (e.g., If they said 5: “What would make you a 1 or 2?”)
2. I’m going to list some things that have been shown to increase your risk of HIV: having sex with multiple partners, not using condoms regularly, sharing syringes/injection equipment, exchanging sex for money or goods, being uncircumcised, engaging in receptive anal sex (which many call being a “bottom”), having a history of sexually transmitted diseases.
   1. Do any of these surprise you?
   2. After hearing this list, would you change your answer to the previous question?
3. Tell me about the last time you got tested for HIV.
   1. Where do you usually go to get HIV testing?
   2. How often do you get tested for HIV?
4. What challenges have you come across in accessing HIV testing?
5. Tell me a bit about your romantic and sexual life?
   1. Tell us about your sexual partners and/or relationships.
   2. Are you currently engaging in sex?
6. What is most important to you when it comes to sexual health?
   1. E.g., safety, earning money, pleasure, etc.
7. How important is it for you to know your sexual partner’s HIV status?
8. Tell me about the last time you talked to your sexual partner about their HIV status?
   1. What was their reaction?
   2. What was your reaction?
9. Do you engage in sexual activities while you are under the influence of alcohol or drugs? If you do, can you tell us about that.
   1. If they say yes: ask them about how much alcohol/drugs they consume
   2. If they say no: ask them what is their understanding of “having sex under the influence of alcohol and drugs”

Knowledge of PrEP:

1. Please tell me what you know about Pre-Exposure Prophylaxis or “PrEP”.
   1. What is it and what is it for?
   2. Who is PrEP recommended for?
2. What is your understanding of the difference between PrEP, PEP (post-exposure prophylaxis), and ART (antiretroviral therapy)?
   1. If they don’t know, explain:
      1. ART is when an HIV-positive person takes anti-HIV medications (antiretrovirals or ARVs) for their lifetime to treat and minimize the effects of HIV.
      2. PrEP is when an HIV-negative person takes an ARV before any possible exposure to HIV to prevent infection.
      3. PEP is when an HIV-negative person takes an ARV soon after a possible exposure to HIV to prevent infection.
3. Tell me some of the things that you have heard people in your community say about PrEP?
   1. What do people in your community think about people who are using PrEP?
4. What is your understanding of how one is supposed to use/take PrEP?
   1. E.g., Route (oral/pill, injectable, patch), Frequency (daily, weekly, …)
5. How much do you think PrEP should cost?
   1. At what price do you think you will be willing to buy and use PrEP?
   2. Do you know where PrEP can be obtained and how much it costs?
6. What have you heard about “on-demand PrEP?” (also called “event-driven PrEP” or “2-1-1”). Please tell me what you know about it.
   1. If never heard of event-driven PrEP: “on-demand PrEP” is when you take PrEP only around the time of anal sex, instead of everyday. It is also known as “2-1-1” because you take 2 pills 2-24 hours before anal sex, 1 pill 24 hours after anal sex, and 1 more pill 48 hours after anal sex.
7. Where did you first learn about on-demand PrEP? Has a healthcare professional (nurse, doctor or pharmacist) ever offered you try this PrEP method?
8. There are potentially many ways that PrEP could be used. We currently use it daily or on-demand, but there are other options being explored: long-acting injections (that last months at a time), rectal inserts, and patches.
   1. What would be your ideal or preferred method of using PrEP?
9. Is there anything that you’d like to get more information on, relating to PrEP or HIV prevention?

Decision Making Around PrEP Use:

1. Have you, yourself, ever taken PrEP? If so, what was that experience like for you?
   1. If you have heard of PrEP before but decided not to use it, can you tell us what contributed to that decision?
   2. If you started using PrEP but then decided to discontinue, can you share why?
2. If you have never taken PrEP: would you be willing to take it? And why?
3. Which method would you prefer more: daily PrEP or on-demand PrEP? And why?
4. How should PrEP services be made available in the community?
   1. How would you prefer to access it? (e.g., clinic (mobile or fixed clinic), community (LGBTQ) organization, pharmacy, online, etc.)
5. What are things that would make it difficult for you to start PrEP and stay on it?
   1. e.g., side effects, medication interactions, financial costs, discomfort discussing with healthcare providers or sexual partner, HIV stigma, sexual stigma
   2. If they self-identify as transgender: If you were taking hormones or gender-affirming medications (e.g. estrogen, testosterone, spironolactone) would that factor into your decision to take PrEP? If so, how?
6. What are some things that would help make it easier for you to start PrEP and stay on it?
   1. e.g., no cost, not a daily pill, less HIV testing, easier to get, acceptance from sexual partner, less side effects, more information/education).
7. Do any of your sexual partners take PrEP? (Currently or in the past).
8. How easy would it be to talk to your relationship partner about PrEP? Why?
9. Would you prefer if your relationship partner was on PrEP? (Maybe instead of you being on PrEP or in addition to you being on PrEP)
10. Tell me how you think your sexual behaviors may change if you start taking PrEP.

Conclusion:

1. Which method of HIV prevention would you most prefer to use and why
   1. e.g., daily PrEp, ED PrEP, long-acting PrEP (injectable, implant), condoms, PEP, treatment as prevention
2. Do you have any other thoughts/comments that you would like to share with us?
